# A commentary on “Tumour immunotherapy: past, present, and future”

**DOI:** 10.1097/JS9.0000000000003441

**Published:** 2025-09-08

**Authors:** Da Huo, Tao He, Yuanling Qi

**Affiliations:** a Department of Traditional Chinese Medicine, Weifang People’s Hospital, Shandong Second Medical University, Weifang, Shandong, China; b Department of Gastroenterology, Weifang People’s Hospital, Shandong Second Medical University, Weifang, Shandong, China

*Dear Editor*,

We read with great interest the recent article titled “Tumour immunotherapy: past, present, and future”^[1]^ published in the *International Journal of Surgery*. An antigen-sensing loop, a cytotoxic-killing loop, an immunoregulatory loop, and a tumor-educating loop are the components of the “synergistic functional loop” model of tumor immunity that is proposed in this paper, which starts with the history and evolution of immunotherapy. However, a number of significant issues need to be addressed in future research.

First and foremost, there is a noticeable deficiency in logical consistency, coupled with a rather disjointed and loosely structured composition throughout the piece. As the narrative unfolds, it attempts to encompass the historical context, the current state, and the prospective advancements in the realm of immunotherapy. However, the inclusion of repetitive content – such as the iterative discussions on vaccine development and the recurring emphasis on the pivotal role of model animals in research – contributes to a sense of structural redundancy that detracts from the overall coherence. Moreover, the transitions between the various sections of the text appear abrupt and disjointed; there exists an absence of a clear and well-defined logical bridge that would effectively connect topics like “vaccine development,” “oncolytic viruses,” and “immunocutaneous therapy” (ICI). This lack of smooth transitions results in a disjointed reading experience, making it exceedingly difficult for readers to maintain a clear understanding and follow the thread of information amidst the overwhelming details. To enhance the readability and comprehensibility of the piece, a more organized approach could be adopted. Specifically, structuring the content around a chronological timeline or a framework that outlines the technological evolution of immunotherapy would be immensely beneficial. Such an approach would not only provide a clearer sense of direction but also enable readers to more easily grasp the developmental trajectory and the progressive advancements within the field of immunotherapy.

Secondly, a notable inconsistency is observed in the manner in which the written explanations and the accompanying charts are integrated within the article. Specifically, when presenting the two central models – the “Synergistic Functional Loop” and the “Efficacy Pyramid” – the corresponding diagrams, namely Figures 1 and 2, are not adequately explained or properly referenced within the main body of the text. This inadequacy creates a significant hurdle for readers, as they struggle to effectively synthesize the textual descriptions with the visual representations provided. Moreover, Tables 1 through 3 present a dense array of data that lacks clear interpretation or a thorough comparative analysis of the essential metrics. This omission substantially diminishes the tables’ utility as a tool to enhance readers’ comprehension, thereby weakening the overall coherence and effectiveness of the article’s explanatory content.

Third, the paper’s references contain missing information and uneven formatting. Some references lack proper citation details (e.g., containing only author and year without journal name, volume/issue, or page numbers), with formatting variations (including some lacking DOI identifiers), which undermines academic rigor. Furthermore, critical conclusions – particularly regarding emerging technologies and clinical failure cases – are supported by insufficient high-quality literature, as evidenced by outdated references or inadequate citations.

While this review demonstrates value in its breadth of content and historical context with valuable conceptual frameworks, it falls short in structural organization, visualization of data, depth of mechanism analysis, technological foresight, practical application considerations, and reference standardization. Future revisions should strengthen logical coherence, deepen mechanism analysis, incorporate discussions on emerging technologies, and standardize reference formatting to enhance both academic rigor and practical utility^[2,3]^. The TITAN guideline, which demands openness in the reporting of artificial intelligence, was mentioned in connection with all of this^[4]^.

## Data Availability

No data was used in this Letter to the Editor.
